# Circulating miR-129-3p in combination with clinical factors predicts vascular calcification in hemodialysis patients

**DOI:** 10.1093/ckj/sfae038

**Published:** 2024-02-15

**Authors:** Jingjing Jin, Meijuan Cheng, Xueying Wu, Haixia Zhang, Dongxue Zhang, Xiangnan Liang, Yuetong Qian, Liping Guo, Shenglei Zhang, Yaling Bai, Jinsheng Xu

**Affiliations:** Departments of Nephrology, The Fourth Hospital of Hebei Medical University, Shijiazhuang, PR China; Hebei Key Laboratory of Vascular Calcification in Kidney Disease, Shijiazhuang, PR China; Hebei Clinical Research Center for Chronic Kidney Disease, Shijiazhuang, PR China; Departments of Nephrology, The Fourth Hospital of Hebei Medical University, Shijiazhuang, PR China; Hebei Key Laboratory of Vascular Calcification in Kidney Disease, Shijiazhuang, PR China; Hebei Clinical Research Center for Chronic Kidney Disease, Shijiazhuang, PR China; Departments of Nephrology, The Fourth Hospital of Hebei Medical University, Shijiazhuang, PR China; Hebei Key Laboratory of Vascular Calcification in Kidney Disease, Shijiazhuang, PR China; Hebei Clinical Research Center for Chronic Kidney Disease, Shijiazhuang, PR China; Departments of Nephrology, The Fourth Hospital of Hebei Medical University, Shijiazhuang, PR China; Hebei Key Laboratory of Vascular Calcification in Kidney Disease, Shijiazhuang, PR China; Hebei Clinical Research Center for Chronic Kidney Disease, Shijiazhuang, PR China; Departments of Nephrology, The Fourth Hospital of Hebei Medical University, Shijiazhuang, PR China; Hebei Key Laboratory of Vascular Calcification in Kidney Disease, Shijiazhuang, PR China; Hebei Clinical Research Center for Chronic Kidney Disease, Shijiazhuang, PR China; Departments of Nephrology, The Fourth Hospital of Hebei Medical University, Shijiazhuang, PR China; Hebei Key Laboratory of Vascular Calcification in Kidney Disease, Shijiazhuang, PR China; Hebei Clinical Research Center for Chronic Kidney Disease, Shijiazhuang, PR China; Departments of Nephrology, The Fourth Hospital of Hebei Medical University, Shijiazhuang, PR China; Hebei Key Laboratory of Vascular Calcification in Kidney Disease, Shijiazhuang, PR China; Hebei Clinical Research Center for Chronic Kidney Disease, Shijiazhuang, PR China; Departments of Nephrology, The Fourth Hospital of Hebei Medical University, Shijiazhuang, PR China; Hebei Key Laboratory of Vascular Calcification in Kidney Disease, Shijiazhuang, PR China; Hebei Clinical Research Center for Chronic Kidney Disease, Shijiazhuang, PR China; Departments of Nephrology, The Fourth Hospital of Hebei Medical University, Shijiazhuang, PR China; Hebei Key Laboratory of Vascular Calcification in Kidney Disease, Shijiazhuang, PR China; Hebei Clinical Research Center for Chronic Kidney Disease, Shijiazhuang, PR China; Departments of Nephrology, The Fourth Hospital of Hebei Medical University, Shijiazhuang, PR China; Hebei Key Laboratory of Vascular Calcification in Kidney Disease, Shijiazhuang, PR China; Hebei Clinical Research Center for Chronic Kidney Disease, Shijiazhuang, PR China; Departments of Nephrology, The Fourth Hospital of Hebei Medical University, Shijiazhuang, PR China; Hebei Key Laboratory of Vascular Calcification in Kidney Disease, Shijiazhuang, PR China; Hebei Clinical Research Center for Chronic Kidney Disease, Shijiazhuang, PR China

**Keywords:** clinical variables, hemodialysis, miR-129-3p, nomogram, vascular calcification

## Abstract

**Background:**

Vascular calcification (VC) commonly occurs and seriously increases the risk of cardiovascular events and mortality in patients with hemodialysis. For optimizing individual management, we will develop a diagnostic multivariable prediction model for evaluating the probability of VC.

**Methods:**

The study was conducted in four steps. First, identification of miRNAs regulating osteogenic differentiation of vascular smooth muscle cells (VSMCs) in calcified condition. Second, observing the role of miR-129–3p on VC *in vitro* and the association between circulating miR-129–3p and VC in hemodialysis patients. Third, collecting all indicators related to VC as candidate variables, screening predictors from the candidate variables by Lasso regression, developing the prediction model by logistic regression and showing it as a nomogram in training cohort. Last, verifying predictive performance of the model in validation cohort.

**Results:**

In cell experiments, miR-129–3p was found to attenuate vascular calcification, and in human, serum miR-129–3p exhibited a negative correlation with vascular calcification, suggesting that miR-129–3p could be one of the candidate predictor variables. Regression analysis demonstrated that miR-129–3p, age, dialysis duration and smoking were valid factors to establish the prediction model and nomogram for VC. The area under receiver operating characteristic curve of the model was 0.8698. The calibration curve showed that predicted probability of the model was in good agreement with actual probability and decision curve analysis indicated better net benefit of the model. Furthermore, internal validation through bootstrap process and external validation by another independent cohort confirmed the stability of the model.

**Conclusion:**

We build a diagnostic prediction model and present it as an intuitive tool based on miR-129–3p and clinical indicators to evaluate the probability of VC in hemodialysis patients, facilitating risk stratification and effective decision, which may be of great importance for reducing the risk of serious cardiovascular events.

## Graphical Abstract

**Graphical Abstract:**
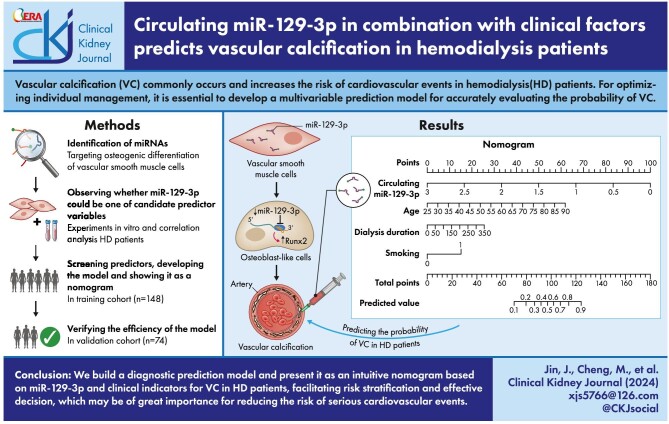

KEY LEARNING POINTS **What was known**:

- In hemodialysis patients, vascular calcification (VC), a common complication, greatly increases the risk of cardiovascular events and mortality.
- However, effective therapies against VC remain unobtainable. For optimizing individual management and improving patient outcomes, it is essential to construct a convenient tool for predicting the probability of VC.

**This study adds**:

- We built a prediction model based on circulating miR-129–3p, age, dialysis duration and smoking, and presented it as a visual nomogram for evaluating the probability of vascular calcification in patients with hemodialysis.

**Potential impact**:

- The model provides a simple and intuitive tool for clinicians to accurately predict the probability of vascular calcification in individual hemodialysis patients, facilitating risk stratification and effective management, and finally reducing the risk of serious cardiovascular events in patients with hemodialysis.

## INTRODUCTION

Vascular calcification (VC), characterized by aberrant deposition of hydroxyapatite crystals in the blood vessel wall, commonly occurs in patients with chronic kidney disease (CKD), especially in those dependent on hemodialysis (HD) [1, 2]. Clinical practice suggests that vascular calcification seriously injures the vessel structure and function and dreadfully increases the chance of cardiovascular events and mortality [3–5]. Recently, despite substantial progress having been made, comprehensive and efficient management for VC is lacking so that the incidence of vascular calcification remains high and rises with dialysis duration and age [6, 7], largely because of failing to identify the risk of VC for individual patients early enough [8]. Therefore, it is of significance to accurately evaluate the possibility of vascular calcification using a diagnostic multivariable model in patients with hemodialysis.

Currently, numerous studies indicate that vascular calcification in hemodialysis patients has an important pathological association with clinical variables including aging, smoking, hypertension, disruption of mineral homeostasis and prolonged duration of dialysis [9–11]. Meanwhile, accumulating evidence has demonstrated that osteogenic differentiation of vascular smooth muscle cells (VSMCs) is the early major pathological change in VC. Thus, besides numerous traditional clinical indicators, it is also essential to screen one stable factor symbolizing osteogenic differentiation of VSMCs when predicting VC in hemodialysis patients.

As is well known, osteogenic differentiation of VSMCs is highly regulated by oxidative stress, cell death, and epigenetic abnormalities [12–14]. MicroRNAs (miRNAs) served as key epigenomic factors and have been discovered to regulate the secretion of various osteogenic differentiation factors, such as runt related transcription factor 2 (Runx2), triggering osteogenic phenotype transition of VSMCs, eventually leading to VC [15, 16]. Nowadays, as noncoding single-stranded RNAs with high stability and superior specificity, miRNAs gradually become excellent biomarkers for relevant diseases [17]. Indeed, previous evidence showed that circulating miRNAs could noninvasively predict vascular calcification more effectively than kidney function eGFR [15]. Moreover, circulating miRNAs have an advantage in forecasting VC progression and deterioration over existing clinical markers [18]. However, it is unclear whether circulating miRNAs united with clinical variables can ameliorate the predictive performance, and no prediction model of miRNAs in combination with clinical indicators has been established to estimate the occurrence of VC in hemodialysis patients.

Therefore, this study sought to construct a diagnostic multivariable prediction model for VC in hemodialysis patients based on miRNA and clinical variables and present it as a nomogram, which was a simple visual tool for clinicians to evaluate the probability of diseases in recent years [19, 20]. To achieve this aim, we identified and included miR-129–3p as one of the VC candidate predictors. Then, all clinical indicators related to VC from the hemodialysis patients were collected and used together with miR-129–3p for the model and nomogram establishment, not only meliorating the efficacy of prediction, but also awarding considerable clinical value.

## MATERIALS AND METHODS

### Study design

The current study strictly followed the Transparent Reporting of a Multivariable Prediction Model for Individual Prognosis or Diagnosis (TRIPOD) [21]. It was designed with four segments. First, by performing bioinformatic analysis, quantitative real-time polymerase chain reaction (qRT–PCR), luciferase assay and RNA-binding protein immunoprecipitation (RIP) assay, miRNAs regulating osteogenic differentiation of VSMCs in calcified condition were screened and identified. Second, the role of miR-129–3p on VC *in vitro* and the association between circulating miR-129–3p and VC in hemodialysis patients was observed to explore whether miR-129–3p could be used as a candidate predictor variable for VC. Third, all clinical indicators related to VC from hemodialysis patients were collected as candidate variables, and predictors were screened from candidate variables by Lasso regression, then the diagnostic prediction model for VC was developed by logistic regression and presented as a nomogram in a training cohort. Last, an independent validation cohort, prospectively collecting data and blood samples, was performed to verify diagnostic efficiency of the model.

### Bioinformatic analysis for miRNAs

The predicted analysis of miRNAs binding to the 3′-untranslated region (3′UTR) of Runx2 mRNA was performed using the miRWalk online program (http://mirwalk.umm.uni-heidelberg.de/), which has the combined power of multiple different programs.

### Cells and experiments *in vitro*

Human arterial smooth muscle cells (ScienCell, no. 6110, San Diego, California, USA), used as VSMCs, were cultured in Smooth Muscle Cell Medium (ScienCell, no. 1101, San Diego, California, USA). Calcification of VSMCs was induced by adding β-glycerophosphoric acid (β-GP, Sigma, Louis, Missouri, USA) to normal medium at a concentration of 10 mM (the calcified condition). HEK293T (293T) cells were obtained from the National Collection of Authenticated Cell Cultures (Shanghai, China). The culture medium was refreshed every two days.

Experiments *in vitro*, including qRT–PCR, miRNA transfection, luciferase reporter assay, RIP assay, immunofluorescence staining, western blot, alizarin red S staining, calcium assay and alkaline phosphatase (ALP) quantification are described in Methods S1 (see online supplementary material).

### Participants

We recruited patients with hemodialysis from the Blood Purification Center in The Fourth Affiliated Hospital of Hebei Medical University, between 1 May 2021 and 30 June 2021, as the training cohort. Additionally, the independent validation cohort was comprised of patients with hemodialysis from another Blood Purification Center in the east courtyard area of The Fourth Affiliated Hospital of Hebei Medical University, between 1 August 2021 and 30 September 2021. Age- and sex-matched healthy individuals were included from the physical examination center of the hospital as the healthy controls. Inclusive and exclusion criteria, sample size calculation and data collection are described in Methods S1 (see online supplementary material). All the participants in this study were Han ethnicity. Written informed consent was obtained from all of the participants. The current study (No. 2020ky189) was approved by the Fourth Affiliated Hospital of Hebei Medical University, and the protocol adhered to the Declaration of Helsinki.

According to coronary artery calcification scores (CACs), hemodialysis participants were categorized as vascular calcification (CACs >0, HD with VC) and non-calcification (CACs = 0, HD without VC) groups. The hemodialysis participants with vascular calcification were divided into mild VC (0<CACs ≤100), moderate VC(100<CACs ≤400), and severe VC(400<CACs) [22].

### Statistical analysis

All statistical analyses were performed using SPSS version 19.0 and R version 4.3.0 software with haven, ggplot2, Matrix, rms, pROC, and ggDCA packages. The sample size was estimated using PASS software v.11. Comparisons between two groups were analysed using the *t*-test, Mann–Whitney *U*, or chi-square test. Comparisons among more than two groups were analysed by one-way analysis of variance (ANOVA). Correlations between two variables were analysed by the Spearman method. Lasso regression was used to determinate the predictors in training cohort. Logistic regression and ablation analysis were used to construct the diagnostic multivariable prediction model and a nomogram was drawn for clinical application. The Harrell concordance index (C-index) analysis, receiver operating characteristic (ROC) curve and area under ROC curve (AUC) were performed to evaluate the predictive ability and discrimination of the model. H–L (Hosmer–Lemeshow) test, calibration curves, and Brier score were conducted to assess the accuracy. Decision curve analysis (DCA) was adopted to estimate clinical net benefit. The model was validated internally using bootstrapping method (100 resamples) and externally on another independent cohort. The two-tailed *P* value <0.05 was considered as a significant difference.

## RESULTS

### MiR-129-3p was found to target the osteogenic differentiation of VSMCs

**Figure 1:**
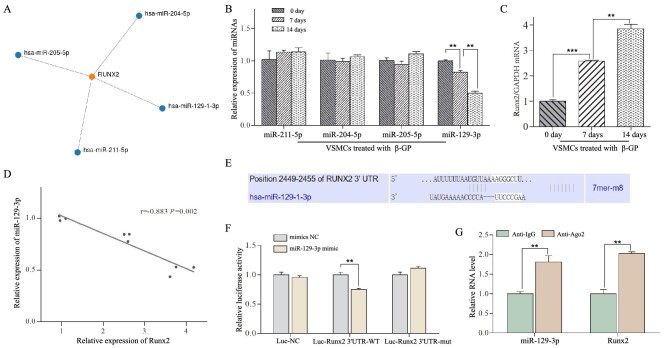
MiR-129–3p was screened and identified to target osteogenic differentiation of VSMCs under calcified conditions. (**A**) Four different miRNAs were predicted to potentially interact with Runx2 mRNA through the miRWalk program. (**B**) The relative expression of the four miRNAs in VSMCs under the calcified medium for 0, 7, and 14 days, suggesting that only miR-129–3p was reduced in a time-dependent manner. **P < 0.01 versus VSMCs treaded with high phosphorus for 0 or 7 days. (**C**) The expression levels of Runx2 mRNA were increased in VSMCs under the calcified medium for 7 and 14 days. ***P < 0.001 and **P < 0.01 versus VSMCs cultured by high phosphorus for 0 or 7 days. (**D**) Negative correlation analysis was revealed between the expression of miR-129–3p and Runx2 in β-GP-induced VSMCs (r = −0.883, *P* = 0.002). (**E**) The sequence of miR-129–3p was predicted to bind Runx2 mRNA. (**F**) Dual-luciferase assay showed that miR‑129–3p mimics significantly decreased the relative luciferase activity in Runx2 3′UTR WT co-transfected cells compared to mimic NC, **P < 0.01. (**G**) Both miR-129–3p and Runx2 mRNA levels were significantly elevated in the precipitant of anti-Ago2 group compared to the anti-IgG group in VSMCs by RIP assays, **P < 0.01.

During vascular calcification, Runx2 acts as an essential osteogenic transcription factor driving the phenotypical switch of VSMCs to osteoblast-like cells [23]. Therefore, in order to detect miRNAs associated with osteogenic differentiation of VSMCs, we used the miRWalk program to predict miRNAs that could be potentially interacted with Runx2. The results showed that four different miRNAs including hsa-miR-129–3p, hsa-miR-204–5p, hsa-miR-205–5p, and hsa-miR-211–5p were discovered through simultaneously filtering with TargetScan and miRDB (Fig. 1A). Through qRT–PCR, we found that only miR-129–3p was decreased in VSMCs under the calcified medium in a time-dependent manner (Fig. 1B). While the Runx2 expression was gradually increased in VSMCs induced by high phosphorus for 7 and 14 days (Fig. 1C), and a negative correlation between miR-129–3p and Runx2 expression was observed (Spearman's r* *=* *−0.883, *P *= 0.002, Fig. 1D). Furthermore, as shown in Fig. 1E, miR-129–3p has potential binding sequences with the 3′UTR of Runx2. The luciferase assay indicated that miR‑129–3p mimics significantly decreased the relative luciferase activity in Runx2 3′UTR WT co-transfected cells compared to mimic NC, but failed to reduce that in Runx2 3′UTR mut plasmids co-transfected cells (Fig. 1F). Similarly, RIP assay also indicated the levels of both Runx2 mRNA and miR-129–3p were significantly elevated in the precipitant of anti-Ago2 group as compared to the anti-IgG group in VSMCs (Fig. 1G). These above results supported that miR-129–3p might target the osteogenic differentiation of VSMCs in calcified medium.

### MiR-129-3p could be used as one of candidate variables for the prediction model of VC

**Figure 2:**
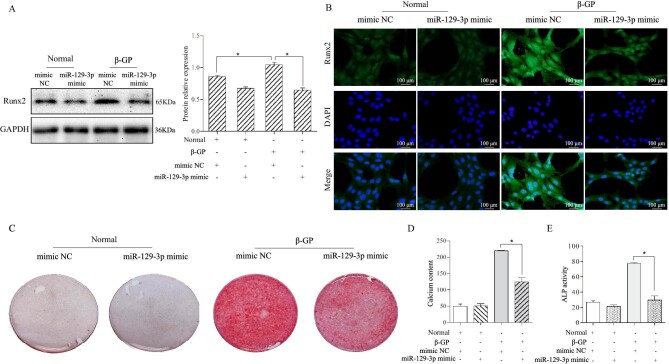
Overexpression of miR-129–3p prevented osteogenic differentiation of VSMCs to alleviate vascular calcification. (**A**) Protein expression levels of Runx2 were determined by western blotting in normal or high phosphorus induced VSMCs transfected with miR‐129–3p mimics or mimic NC, *P < 0.05. (**B**) Immunohistochemical staining of Runx2 was performed in each group. (**C**) VSMCs were cultured and stained with Alizarin Red S for calcium deposition. (**D**) Calcium contents of VSMCs with same treatment as stated above were assessed using a calcium assay kit, *P < 0.05. (**E**) ALP activity of VSMCs was measured in each group, *P < 0.05.

To assess the role of miR-129–3p in VSMCs under calcified condition, we constructed miR-129–3p mimics. After transfection with miR-129–3p mimics, western blot revealed that the ability of high phosphorus to provoke Runx2 expression was obviously inhibited (Fig. 2A). Consistently, overexpression of miR-129–3p significantly reduced the immunofluorescence level of Runx2 in VSMCs incubated with high phosphorus stimulation for seven days (Fig. 2B). Furthermore, miR-129–3p mimics alleviated red calcium deposition in VSMCs treated with high β-GP medium for seven days (Fig. 2C). Meanwhile, both calcium content and ALP activity also confirmed that miR-129–3p overexpression protected VSMCs against calcification under high β-GP medium (Fig. 2D and E). Together, these data indicated that miR-129–3p could alleviate VSMCs calcification induced by high phosphorus.

**Figure 3:**
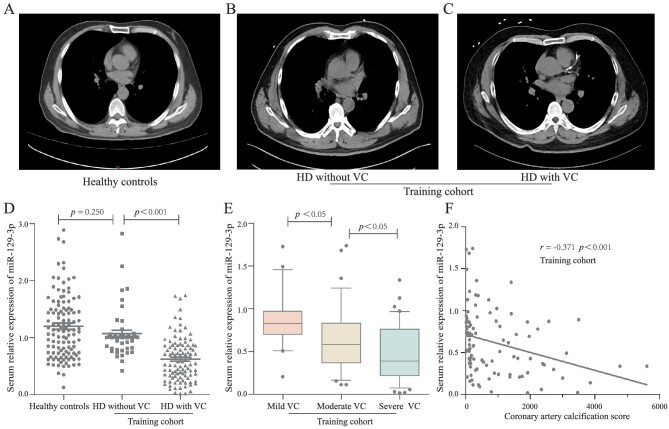
The association between circulating miR-129–3p and vascular calcification in HD patients. (**A**–**C**) Radiological quantification of coronary artery calcification using dual-source computed tomographic scanner in healthy controls, HD patients without VC and HD patients with VC from training cohort, respectively. (**D**) Serum levels of miR-129–3p in healthy controls, HD patients without VC and HD patients with VC from training cohort. (**E**) Serum levels of miR-129–3p in HD participants with mild VC, moderate VC, and severe VC from training cohort. (**F**) The negative correlation analysis between serum miR-129–3p and coronary artery calcification scores in HD patients with VC from the training cohort.

Furthermore, we observed the association between circulating miR-129–3p and VC in hemodialysis patients. In this study, 148 hemodialysis patients were enrolled as the training cohort, which could meet the sample size requirement (power = 0.9588). And 116 age- and gender-matched healthy individuals without vascular calcification were included as the healthy controls. Baseline characteristics of all participants are shown in Table S1 (see online supplementary material). In the training cohort, the incidence of vascular calcification was 68.9%, which was high and consistent with the previous reports [24, 25]. The computed tomography images assessing coronary artery calcification are displayed in Fig. 3A–C. Subsequently, qRT-PCR was manipulated to detect circulating miR-129–3p levels. As shown in Fig. 3D, compared with the healthy samples and HD patients without VC, circulating miR-129–3p were statistically reduced in HD patients with VC (*P* < 0.001), which exhibited the same trend within the above results *in vitro*. The expression of miR-129–3p varied with mild, moderate and severe VC, exhibiting a gradual downward trend in serum (Fig. 3E). Moreover, a directly negative correlation between miR-129–3p and patients’ CACs was revealed by Spearman analysis (r = −0.371, *P*<0.001, Fig. 3F). Overall, miR-129–3p could be one of the candidate variables for the diagnostic prediction model of VC.

### Screening predictors from the candidate variables for building the model in the training cohort

**Figure 4:**
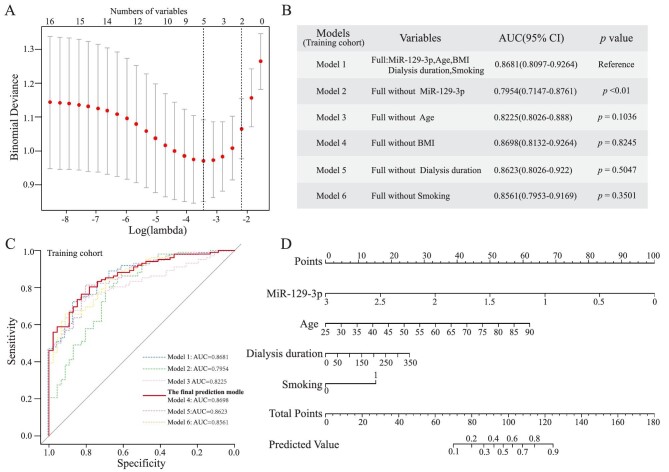
Development of the prediction model and nomogram for vascular calcification in HD patients in the training cohort. (**A**) LASSO regression by ten-fold cross-validation method showed the optimal lambda was 0.0318, and five variables were selected as candidate predictors for further logistic regression analysis. (**B**) Results of the logistic regression and ablation analysis, showing that model 4 with the best satisfactory predictive discrimination was identified as the final predict model. (**C**) ROC curves for models; the solid line denotes the final prediction model. (**D**) Nomogram for the final prediction model of vascular calcification in HD patients.

To pick out the most predictable variables for model construction, we collected clinical characteristics between calcification and non-calcification groups (Table 1) and employed the Lasso regression to filter the candidate variables, including miR-129–3p and clinical variables such as age, sex, smoking history, BMI, hypertension, dialysis duration, sKt/v, SCr, hemoglobin, albumin, cholesterol, potassium, phosphate, corrected serum calcium, iPTH, vitamin D, and ALP. Ten-fold cross-validation was used to select the optimal model. The results showed that the optimal lambda was 0.0318 when the error of model was minimized, and miR-129–3p and four clinical variables, including age, dialysis duration, smoking, and BMI were chosen as predictors of VC for further logistic regression analysis (Fig. 4A).

**Table 1:** Baseline characteristics of patients with or without vascular calcification in the training cohort and the validation cohort.

	Training cohort			Validation cohort		
Characteristics	HD without vascular calcification (*n* = 46)	HD with vascular calcification (*n* = 102)	*P*-value	HD without vascular calcification (*n* = 25)	HD with vascular calcification (*n* = 49)	*P*-value
Age, y	44.4 ± 13.2	57.9 ± 13.2	P<0.001	47.8 ± 18.2	57.9 ± 15.4	*P *= 0.015
Male, %	25 (54.3%)	64 (62.7%)	*P *= 0.368	12 (48.0%)	30 (61.2%)	*P *= 0.326
Smoker, %	9 (19.6%)	32 (31.4%)	*P *= 0.167	3 (12.0%)	13 (26.5%)	*P *= 0.233
BMI (kg/m^2^)	22.2 ± 4.8	21.8 (5.7)	*P *= 0.817	23.7 ± 4.6	22.0 ± 3.7	*P *= 0.120
Hypertension, %	37 (80.4%)	93 (91.2%)	*P *= 0.064	23 (92.0%)	44 (89.8%)	*P *= 0.759
Dialysis duration, m	69.9 ± 50.7	97.1 ± 65.0	*P *= 0.027	69.0 ± 60.5	104.5 ± 60.9	*P *= 0.045
sKt/v	1.4 (0.30)	1.3 (0.18)	*P *= 0.211	1.3 (0.21)	1.3 (0.24)	*P *= 0.673
Hemoglobin, g/L	111.2 ± 12.9	113.0 (11.7)	*P *= 0.604	114.4 ± 9.4	113.9 ± 9.9	*P *= 0.829
Albumin, g/L	41.1 (5.0)	39.9 ± 3.0	*P *= 0.225	42.3 (5.9)	39.4 ± 4.2	*P *= 0.053
Cholesterol, mmol/L	4.90 ± 0.93	4.50 ± 1.11	*P *= 0.148	4.66 ± 1.01	4.76 ± 1.05	*P *= 0.796
Potassium, mmol/L	4.9 ± 0.71	4.8 ± 0.67	*P *= 0.919	5.1 ± 0.50	4.8 ± 0.76	*P *= 0.078
Phosphate, mmol/L	1.5 ± 0.62	1.5 ± 0.51	*P *= 0.854	1.6 ± 0.56	1.5 ± 0.60	*P *= 0.751
Calcium, mmol/L	2.19 ± 0.20	2.18 (0.27)	*P *= 0.286	2.25 ± 0.15	2.23 ± 0.21	*P *= 0.757
iPTH, pg/mL	163.0 (155.5)	174.9 (163.1)	*P *= 0.270	190.9 (189.8)	167.8 (186.0)	*P *= 0.373
Vitamin D, ng/mL	15.5 (10.6)	15.5 (9.7)	*P *= 0.785	16.0 (10.4)	14.6 (8.4)	*P *= 0.507
ALP, U/L	86.7 (46.2)	93.9 (50.8)	*P *= 0.622	86.7 (40.7)	94.0 (46.0)	*P *= 0.664

### Development of the prediction model and nomogram for VC in HD patients

Logistic regression model was established based on the above five predictors (Model 1). To appraise the contribution of each indicator, we conducted a model ablation analysis, removing miR-129–3p, age, BMI, dialysis duration, and smoking in turn and watching the changes in predictive efficacy. As shown in Fig. 4B, removing any variable except BMI would decrease the AUC of the model, which indicated that miR-129–3p, age, dialysis duration, and smoking played essential roles in the model, especially the absence of miR-129–3p resulting in a significant decline of the AUC from 0.8681 to 0.7954 (*P* < 0.01). Therefore, the final prediction model was built based on miR-129–3p, age, dialysis duration, and smoking (Model 4) with satisfactory predictive discrimination, which had an AUC of 0.8698 (Fig. 4C). The predicted probability of vascular calcification in patients with hemodialysis was determined by logit (p) = −1.6927–2.6843 × miR-129–3p + 0.0769 × age + 0.0059 × dialysis duration + 1.2251 × smoking.

Then, a nomogram was presented based on the final model (Fig. 4D). For an individual HD patient, higher total scores revealed a higher risk of vascular calcification. For example, if a patient was 70 years old, on hemodialysis for 5 years (60 months), smoking, and if his serum relative expression of miR-129–3p was 1.5, then the corresponding points would be approximately 42.7, 4.2, 15.1, 50, respectively. The total points were approximately 112, suggesting an estimated vascular calcification of 77% for this case.

### Predictive accuracy and net benefit of the prediction model

**Figure 5:**
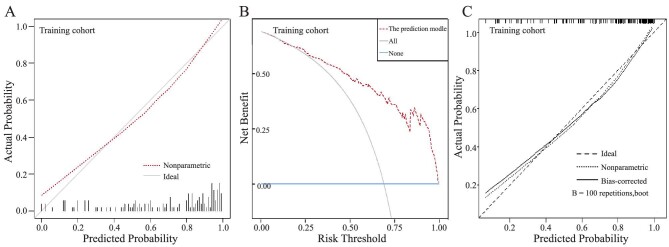
Predictive accuracy and net benefit of the model. (**A**) Calibration curve for the prediction model in training cohort. (**B**) The decision curve analysis of the prediction model in training cohort. (**C**) The internal validation calibration curve by bootstrap process in training dataset.

In the training cohort, the calibration curve indicated that predicted probability was in good agreement with actual probability of VC (Fig. 5A). Meanwhile, the H–L test exhibited a nice fitting degree of the model (*P*>0.05); and the Brier score was 0.137, suggesting that the model had predictive accuracy. Furthermore, the DCA demonstrated that the model delivered better net benefits (Fig. 5B). Through bootstrap process, internal validation yielded an optimism-corrected C-index of 0.8534 and the mean absolute error between bias-corrected calibration curve and nonparametric curve was only 0.029 (Fig. 5C).

### Validation of the prediction model by another independent cohort

**Figure 6:**
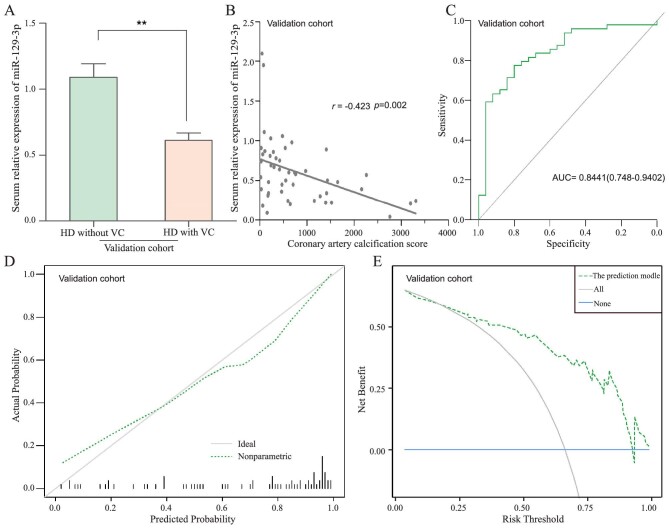
Validation of the prediction model by another independent cohort. (**A**) Serum levels of miR-129–3p in HD patients without VC were higher than that in HD patients with VC from the validation cohort, **P < 0.01. (**B**) The negative correlation analysis between serum miR-129–3p and coronary artery calcification scores in HD patients with VC from the validation cohort. (**C**) ROC for the prediction model in the validation cohort. (**D**) The calibration curve of the prediction model in the validation cohort. (**E**) The decision curve analysis of the prediction model in the validation cohort.

In the validation cohort, 74 HD patients from east courtyard area of the Fourth Affiliated Hospital of Hebei Medical University were used to confirm the prediction model. As shown in Fig. 6A and B, patients with higher CAC scores were accompanied by lower levels of miR-129–3p, similar to that in the training cohort. Furthermore, applying identical logistic formula to the validation cohort, a new ROC curve was built and the AUC was 0.8441 (Fig. 6C), displaying a good consistency with the training cohort. Meanwhile, through external validation, the calibration curve was close to the ideal diagonal, and the Brier score was 0.149 (Fig. 6D), reflecting that the prediction model had good calibration. Moreover, similar results of significant clinical usefulness were found by the DCA in the validation cohort (Fig. 6E). Overall, these outcomes revealed the robustness and application potential of the prediction model for clinical decision making.

## DISCUSSION

In this study, we have for the first time constructed a combined miRNA and clinical variables model to predict the probability of vascular calcification in hemodialysis patients, although several reports about vascular calcification prediction focused only on miRNAs [15, 26] or clinical factors [27, 28], respectively. Based on miR-129–3p, age, dialysis duration, and smoking, the diagnostic multivariable prediction model exhibited good discrimination and calibration and was presented as a simple, intuitive nomogram to calculate the probability of vascular calcification in every patient with hemodialysis.

As is well known, with superior specificity and high stability, circulating miRNAs have been investigated as excellent noninvasive biomarkers to monitor different diseases, such as cardiovascular diseases and cancers [29, 30]. Currently, relevant studies have revealed important roles of several miRNAs in the development and progression of vascular calcification through modulating VSMCs osteogenic differentiation [31]. Our previous studies showed [32, 33] that Runx2 was a core factor of multiple pathways leading to VC under calcified conditions, as it acted as an osteogenic transcription marker to trigger the expression of osteogenic transcriptome genes in VSMCs [34–36]. Therefore, we used Runx2 to explore the miRNAs for building the prediction model. In our findings, miR-129–3p was exhibited to involve osteogenic differentiation of VSMCs by targeting the Runx2 3′UTR region. Similarly, Li *et al.* also showed that miR‐129‐3p regulated endothelial progenitor cell differentiation and angiogenesis through Runx2 [37]. Moreover, we discovered that miR‐129‐3p overexpression reduced VSMCs calcification induced by high phosphorus, which was consistent with recent results [38] showing that accumulation of miR-129–3p decreased mineralized nodule formation in diabetic periodontal tissue. Furthermore, accordant with the results presented *in vitro*, we found circulating miR‐129‐3p was negatively correlated with the severity of calcification. And compared with other clinical variables, if miR‐129‐3p was removed, the differentiation degree of the model decreased most significantly. Thus, these results demonstrated that miR-129–3p was a very suitable and essential member in the diagnostic prediction model for vascular calcification.

Additionally, according to emerging research, vascular calcification is also a hallmark of aging [7]. With advancing age, even individuals without CKD gradually present vascular lesions including arterial calcification and stiffness, increasing the risk of cardiovascular disease [13]. Moreover, accumulating studies used animal models resembling the aging process have demonstrated that VSMCs senescence and oxidative stress were major determinants of age-associated vascular calcification [39–41]. However, there are no currently available therapies specifically preventing age-associated vascular dysfunction. In view of this, it is necessary to use age as an effective predictor for evaluating the probability of vascular calcification to stratify management in a more timely fashion, reducing the risk of serious cardiovascular events. In this study, the age of patients in both the training and validation cohorts was significantly higher in the calcification group and was included in the predictive model. Moreover, when age was removed, the discrimination power of the model would be reduced from 0.8661 to 0.8225, reflecting that age was another important factor in the accurate prediction of the risk of vascular calcification.

The third independent predictor of vascular calcification in the current study was the dialysis duration. Because it is removing the majority of the volume in a short time, hemodialysis leads to overt fluctuations in blood pressure, resulting in cell lesions and inflammation, eventually increasing the occurrence of detectable VC after a period of accumulation. Really, Chen *et al.* reported that dialysis duration of more than five years raised the prevalence of radial artery calcification [42], and Xiong *et al.* found that HD patients with CAC were more likely to have a longer duration of dialysis [6]. Consistent with the above studies, our study found that the dialysis duration of patients with calcification was significantly prolonged, and adding dialysis duration into the model would improve the predictive power, suggesting a synergistic predictive effect of dialysis duration with other indicators on vascular calcification.

Smoking was also an independent risk factor of vascular calcification. Although without significant difference, our data showed that the rate of tobacco use in the calcification group was higher than that of patients in the non-calcification group (19.6% vs 31.4%). Moreover, smoking was screened by lasso regression and played an indispensable part in our prediction model. A prospective study in Korean also demonstrated that compared to never smokers, the prevalence of CAC was 1.25-fold higher in current smokers, and long-term smoking cessation would reduce the risk of CAC [43]. Therefore, it was worthy to take smoking into the diagnostic multivariable model for facilitating risk management.

In this study, for the clinical translation, we visualized the prediction model as a nomogram with good efficiency and accuracy, as well as better net benefit. Previously, although several studies have also attempted to construct prediction models of vascular calcification, most of these studies were short of comprehensive consideration of predictors, and more importantly none of these studies had external validation or provided convenient visual tools [44, 45]. Certainly, there are several limitations in our study. Firstly, we did not include diabetics in our analysis, thus the prediction model may not be appropriate for diabetes. Secondly, some variables related to mineral metabolism, such as phosphate, calcium, iPTH, were not included into the model, but this was because drug treatment in clinical practice made these indices have no difference between the calcification and non-calcification groups. Finally, serum miR-129–3p and clinical variables in our cohorts were only representative of the Hebei region in China; therefore, further nationwide external validation research is needed to verify the reliability of the model.

In conclusion, we construct a diagnostic multivariable prediction model based on miR-129–3p and clinical variables including age, dialysis duration, and smoking, and present it as a simple and intuitive nomogram to evaluate the probability of vascular calcification for individual hemodialysis patient, facilitating risk stratification and effective management, which may be of great importance for reducing the risk of serious cardiovascular events.

## Supplementary Material

sfae038_Supplemental_Files

## Data Availability

All data generated or analysed during this study are included in this article. Further inquiries can be directed to the corresponding author.
