# Correction to: Circulating miR-129-3p in combination with clinical factors predicts vascular calcification in hemodialysis patients

**DOI:** 10.1093/ckj/sfaf300

**Published:** 2025-10-21

This is a correction to: Jingjing Jin, Meijuan Cheng, Xueying Wu, Haixia Zhang, Dongxue Zhang, Xiangnan Liang, Yuetong Qian, Liping Guo, Shenglei Zhang, Yaling Bai, Jinsheng Xu, Circulating miR-129-3p in combination with clinical factors predicts vascular calcification in hemodialysis patients, *Clinical Kidney Journal*, Volume 17, Issue 3, March 2024, sfae038, https://doi.org/10.1093/ckj/sfae038

The authors have recently identified an error in the supplemental file entitled ‘Supplementary Materials and Methods’, which accompanies the originally published version of this manuscript.

In the section ‘Sample size calculation’**,** the Odds Ration (OR) value from the pre-experiment should have been stated as 0.517, instead of 0.65, in the following sentence:

“Odds Ratio was set as 0.65 by pre-experiment of serum miR-129-3p, R-Squared was 0.15, and the 2-way type I error was 5%, we estimated that the sample size of 113 patients would have a 90% power to develop the model with a very good fit.”

This sentence should correctly read:

“Odds Ratio was set as 0.517 by pre-experiment of serum miR-129-3p, R-Squared was 0.15, and the 2-way type I error was 5%, we estimated that the sample size of 113 patients would have a 90% power to develop the model with a very good fit.”

These details have been corrected only in this correction notice to preserve the published version of record.

