# Supplementary material for: Circulating miR-129-3p in combination with clinical factors predicts vascular calcification in hemodialysis patients
Source: Clin Kidney J. 2024 Feb 15;17(3):sfae038. doi: 10.1093/ckj/sfae038 (PMC10960567; doi:10.1093/ckj/sfae038)
Supplement: sfae038_Supplemental_Files [file sfae038_supplemental_files.zip › Supplementary Methods.docx]

**Supplementary Materials and Methods**

**Quantitative real-time polymerase chain reaction (****qRT–PCR)**

Total RNA was extracted from VSMCs or serum with TRIzol reagent, and then synthesized cDNA by a commercial miRNA reverse transcription PCR kit (RiboBio, Guangzhou, China) and a PrimeScript™ RT reagent Kit (Takara, Japan) for mRNA following the manufacturer's protocol. Quantitative PCR of miRNAs (has-miR-129-3p, has-miR-204-5p, has-miR-205-5p, has-miR-211-5p) was performed using a miDETECT A Track™ miRNA qRT–PCR Kit (RiboBio, Guangzhou, China), and quantitative PCR of Runx2 mRNA was performed using a TB Green® Premix Ex Taq™ II (Takara, Japan). U6 small nuclear or GAPDH was chosen as an endogenous control for miRNA or mRNA in VSMCs. For serum miR-129-3p detection, *Caenorhabditis elegans* miR-39-3p was added to the isolated RNAs before cDNA synthesis and used as an exogenous control. Relative expression levels of the miRNAs were calculated using the 2^−ΔΔCt^ method.

**miRNA transfection**

The miRNAs were purchased from Zhongshi Gene Technology Co., Ltd (Tianjin, China), and the sequences were as follows: miR-129-3p mimic, AAGCCCUUACCCCAAAAAG; mimic negative control (mimic NC), UUGUACUACACAAAAGUACUG. Cell transfection was carried out by a Lipofectamine ^®^ 3000 Transfection Kit following manufacturer’s instructions (Invitrogen, California, USA). The miR-129-3p mimic and mimic NC were transfected into 293T cells or VSMCs. Then, VSMCs were harvested and tested after transfection 48 h.

**Luciferase reporter assay**

Dual luciferase reporter plasmids carrying Runx2 3′UTR wild-type (WT) or mutant (mut) fragments containing putative binding sites for miR-129-3p (Luc-Runx2 3′UTR-WT/Luc-Runx2 3′UTR-mut) and luciferase reporter plasmids negative control (Luc-NC) were obtained from Zhongshi Gene Technology Co., Ltd (Tianjin, China). The plasmids were co-transfected with miR-129-3p mimic or mimic NC into the 293T cells, respectively. After transfection 48 h, luciferase assays were conducted using [Dual-Glo® Luciferase Assay System](https://www.promega.com.cn/products/luciferase-assays/reporter-assays/dual_glo-luciferase-assay-system/?catNum=E2920) (Promega Corporation, Madison, WI, USA). The relative luciferase activity value was presented as ratios of firefly luciferase activity against Renilla luciferase activity.

**RNA-binding protein immunoprecipitation (RIP)** **assay**

An EZ-Magna RIP™ RNA-binding protein immunoprecipitation kit was obtained from Millipore Co., Ltd (Billerica, MA, USA) to perform the RIP assay according to the manufacturer’s protocols. Briefly, VSMCs was lysed, centrifuged and collected supernatant. Then the liquid was pre-precipitated with magnetic beads bound to an anti-Ago2 antibody or anti-IgG antibody, Millipore, Billerica, MA, USA). Finally, co-precipitated RNAs were extracted and reversely transcribed to detected runx2 and miR-129-3p levels using qRT–PCR analysis.

**Immunofluorescence staining**

VSMCs were fixed with 4% paraformaldehyde for 20 min and permeabilized with 0.2% Triton X-100 (Beijing Solarbio, China) for 30 min on coverslips in 24-well plates. After blocking with 5% normal goat serum, they were followed by incubation at 4 °C overnight with anti-Runx2 primary antibody (ab192256, Abcam, MA, USA). Then, Dylight 488 conjugated goat anti-rabbit IgG (A23220, Abbkine, California, USA) was served as secondary antibody to incubate the cells at room temperature for 60 min. Finally, they were observed with a fluorescence microscope after coloration with DAPI (Sigma, St. Louis, MO, USA) for another 10 min to detect Runx2 (green) and DAPI (blue) in the VSMCs.

**Western blot**

Western blot was operated as previously described [1]. Protein extracted from VSMCs using lysis buffer were electrophoresed on SDS-PAGE gel and electrotransfered onto polyvinylidene difluoride membranes. The membranes were incubated with primary antibodies at 4°C overnight. The primary antibodies included anti-Runx2 antibody and anti-GAPDH antibody (ab8245, Abcam, MA, USA). Then, the membranes were incubated with relative secondary antibody at room temperature for two hours. Pierce ECL reagent (Thermo Fisher Scientific, MA, USA) and Image ProPlus v.5.0 software (Media Cybernetics, Inc. USA) were used to visualize and measure integrated optical density (IOD) of immunoblots on the membranes. Finally, the protein relative expression levels were presented as ratios of Runx2/GAPDH IOD.

**Alizarin red S staining，Calcium assay and** **alkaline phosphatase (ALP) quantification**

For Alizarin Red S staining, VSMCs were fixed with 70% ethanol in six-well plates and then stained with 1 mg/ml Alizarin Red S solution (Servicebio, China) for 60 min at room temperature. The calcium deposits were observed under a microscope.

VSMCs were decalcified with hydrochloric acid for 12h at 4°C. Then calcium concentration in the supernatant was detected using a calcium content kit (Nanjing Jian cheng Bioengineering Institute, China) according to the manufacturer’s protocols.

A commercial kit was obtained from Nanjing Jian cheng Bioengineering Institute to determine ALP activity of VSMCs following the manufacturer’s instructions. The results were calculated by the standard curves.

**Inclusive and exclusion criteria for** **patients with** **hemodialysis**

Inclusive criteria were as follows: age ≥ 18 years old; available various clinical indicators data; stable hemodialysis for at least three months (three times a week for four hours each time). Exclusion criteria were as follows: with serious coexisting illnesses requiring intensive care, diabetes mellitus, autoimmune diseases, malignant tumor, and organ transplantation.

**Sample size calculation**

According to previous research, revealing 55% to 77.4% of dialysis patients have occurred coronary artery calcification[2, 3], we estimated at least a 50% rate of coronary artery calcification in our patients. Odds Ratio was set as 0.65 by pre-experiment of serum miR-129-3p, R-Squared was 0.15, and the 2-way type I error was 5%, we estimated that the sample size of 113 patients would have a 90% power to develop the model with a very good fit. In order to account for expected losses records, we need to oversample by 10% of the estimated sample size. Finally, the minimum sample size was 124 for training cohort in our study.

In our training cohort, the clinical information of total 339 patients were obtained from Blood Purification center in The Fourth Affiliated Hospital of Hebei Medical University; 191 did not meet the inclusion and exclusion criteria, of which 127 patients combined with diabetes and 64 patients had missing some clinical data, including CACs, albumin, cholesterol, etc. Finally, 148 patients were enrolled as the training cohort in this study, which could meet the sample size requirement (power = 0.9588).

**Data Collection**

Fasting blood samples were obtained from healthy control and HD patients before first-week dialysis sessions. The blood sample was centrifuged at 400g for 20 minutes and 200ul of supernatant was collected as serum for RNA extraction. The collected data from enrolled participants comprised the general conditions (age, sex, smoking history, body mass index, comorbidities, dialysis duration, and sKt/v), clinical biochemical variables (serum creatinine (SCr), hemoglobin, albumin, cholesterol, and potassium), other clinical factors associated with calcium-phosphorus metabolism (phosphate, corrected serum calcium, intact parathyroid hormone (iPTH), vitamin D, and ALP), and serum miR-129-3p. A 128-row multi-detector dual-source computed tomographic scanner was used to evaluate coronary artery calcification. Scans were analyzed using SmartScore software (Definition Flash, Siemens Healthcare, Forchheim, Germany), and the total coronary artery calcification score (CACs) was calculated according to the Agatston method.

1. Xu J, Bai Y, Jin J*, et al.* Magnesium modulates the expression levels of calcification-associated factors to inhibit calcification in a time-dependent manner. Exp Ther Med 2015;9(3):1028-1034

2. Huang Y, Ge Y, Li F*, et al.* Elucidating the relationship between nutrition indices and coronary artery calcification in patients undergoing maintenance hemodialysis. Ther Apher Dial 2022;26(1):71-84

3. Liu ZH, Yu XQ, Yang JW*, et al.* Prevalence and risk factors for vascular calcification in Chinese patients receiving dialysis: baseline results from a prospective cohort study. Curr Med Res Opin 2018;34(8):1491-1500
