# Supplementary material for: Circulating miR-129-3p in combination with clinical factors predicts vascular calcification in hemodialysis patients
Source: Clin Kidney J. 2024 Feb 15;17(3):sfae038. doi: 10.1093/ckj/sfae038 (PMC10960567; doi:10.1093/ckj/sfae038)
Supplement: sfae038_Supplemental_Files [file sfae038_supplemental_files.zip › Supplementary Table 1.docx]

| **Characteristics** | **Healthy controls (n=****116)** | **Hemodialysis patients** | | |  |
| --- | --- | --- | --- | --- | --- |
|  |  | **Training cohort (n =****148)** | **Validation cohort (n =****74)** | **Total (n=222)** |  |
| Age, y | 52.0±11.1 | 53.7±14.6 | 54.5±17.0 | 54.0±15.4 |  |
| Male, % | 61(51.7%) | 89(60.1%) | 42(56.8%) | 131(59.0%) |  |
| Smoker, % | 22(18.6%) | 41(27.7%) | 16(21.6%) | 57(25.7%) |  |
| BMI (kg/m^2^) | 23.1±3.2 | 22.4±4.2 | 22.6±4.1 | 22.4(6.0) |  |
| Hypertension, % | 25(21.1%) | 130(87.8%) ^b,c^ | 67(90.5%) ^b,c^ | 197(88.7%) ^b,c^ |  |
| Dialysis duration, m | / | 82.4(97.1) | 76.8(112.4) | 80.5(100.7) |  |
| sKt/v | / | 1.3(0.19) | 1.3(0.20) | 1.3(0.19) |  |
| Scr, umol/L | 62.5±13.4 | 919.7±248.3 ^b,c^ | 1004.0(346.0) ^b,c^ | 943.3±235.1 ^b,c^ |  |
| Hemoglobin, g/L | 144.5(25.3) | 113(13.5) ^b,c^ | 114.1±9.6 ^b,c^ | 113.0(12.2) ^b,c^ |  |
| Albumin, g/L | 44.8(2.7) | 40.1±3.2 ^b,c^ | 40.0±3.9 ^b,c^ | 40.5(4.6) ^b,c^ |  |
| Cholesterol, mmol/L | 4.9±0.8 | 4.7±1.1 | 4.7±1.0 | 4.7±1.0 |  |
| Potassium, mmol/L | 3.9(0.5) | 4.8±0.7 ^b,c^ | 4.9±0.7 ^b,c^ | 4.8(0.9) ^b,c^ |  |
| Phosphate, mmol/L | 1.35±0.26 | 1.52±0.54 ^a,c^ | 1.55±0.58 ^a,c^ | 1.47(0.66) ^a,c^ |  |
| Calcium, mmol/L | 2.21(0.14) | 2.17(0.26) | 2.23(0.28) | 2.20(0.26) |  |
| iPTH, pg/mL | 39.7(14.1) | 169.5(153.9) ^b,c^ | 182.3(177.2) ^b,c^ | 170.8(183.1) ^b,c^ |  |
| Vitamin D, ng/mL | 17.9(8.8) | 15.5(10.4) ^b,c^ | 14.8(9.6) ^a,c^ | 15.4(9.7) ^b,c^ |  |
| ALP, U/L | 89.5±42.8 | 91.4(49.9) ^a,c^ | 92.4(43.8) ^a,c^ | 92.0(47.5) ^a,c^ |  |

**Supplementary Table 1.** Baseline characteristics of all participants

^a^ Significant at p value <0.05.

^b^ Highly significant at p value <0.001.

^c^ Significant difference versus healthy controls.
